# Organizing pneumonia associated with T‐cell lymphoma

**DOI:** 10.1002/rcr2.677

**Published:** 2020-10-26

**Authors:** Chrystal Chan, Andrei D. Fagarasanu, Andre Reid, Daniel C. Sadowski, Ashley‐Mae E. Gillson

**Affiliations:** ^1^ Department of Medicine University of Alberta Edmonton AB Canada; ^2^ Department of Laboratory Medicine & Pathology University of Alberta Edmonton AB Canada

**Keywords:** Lung biopsy, lymphoma, organizing pneumonia

## Abstract

A 61‐year‐old male with a history of coeliac disease was diagnosed with organizing pneumonia (OP) on transbronchial and transthoracic lung biopsies. He then developed refractory coeliac disease type II and haemophagocytic lymphohistiocytosis. Nine months after his initial diagnosis of OP and after multiple biopsies of the lung, duodenum, and bone marrow, he was diagnosed with enteropathy‐associated T‐cell lymphoma (EATL). Although OP in patients with lymphoma is most commonly attributed to chemotherapeutic agents or bone marrow transplant, it may be seen in the absence of prior anticancer treatment. The mechanism linking OP and lymphoma is unclear but OP could represent a syndrome of T‐cell dysfunction or develop as a direct reaction to malignant infiltration of the lung. In patients with atypical presentations, exclusion of an alternate diagnosis must be pursued using surgical lung biopsy, wherever possible. This is the first reported case of OP associated with EATL.

## Graphical abstract

We report a rare case of organizing pneumonia (OP) associated with T‐cell lymphoma without prior anticancer therapy. OP could represent a syndrome of T‐cell dysfunction or develop as a direct reaction to malignant infiltration of the lung. In patients with atypical presentations, exclusion of an alternate diagnosis must be pursued using surgical lung biopsy, wherever possible.

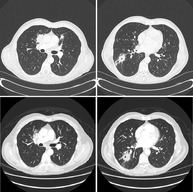

## Introduction

Organizing pneumonia (OP) is a clinicopathological entity resulting from an aberrant reparative response to lung injury. Patients typically present with variable degrees of dyspnoea, cough, malaise, and fever over several months. OP is most commonly characterized on high‐resolution computed tomography (HRCT) by bilateral, patchy, often migratory consolidation in a subpleural, peribronchial, or bandlike pattern, frequently associated with ground‐glass opacities. Histopathology is characterized by fibroblast plugs within the small airways, often extending into adjacent alveoli. OP is typically very steroid‐responsive, however, relapse is common [1, 2].

OP is most often idiopathic and labelled cryptogenic OP [1]. Secondary OP is associated with a variety of causes including infection, immunological disorders, drugs, radiation injury, and bone marrow transplantation. OP is seen frequently in patients with lymphoma; however, in these cases, it is usually attributed to anticancer treatment. Numerous chemotherapeutic drugs have been implicated in OP, including methotrexate, bleomycin, doxorubicin, and rituximab [1, 2, 3]. Many of these agents constitute first‐line therapy for lymphoma, making it difficult to find cases where OP is associated directly with the malignancy itself. Attributable respiratory mortality remains low at 14% in patients with haematological malignancies and OP [4], although prognosis is worse than that of cryptogenic OP, which has a mortality rate of 5% [1, 5].

We present the first case of OP associated with enteropathy‐associated T‐cell lymphoma (EATL). Given the increasing incidence of OP and the growing number and increasing efficacy of antineoplastic treatments for lymphoma, elucidation of a relationship between OP and lymphoma is crucial.

## Case Report

A 61‐year‐old male, previously well, was diagnosed with localized ileocaecal adenocarcinoma in September 2016 after presenting with a positive faecal immunochemical test on routine screening. He underwent a right hemicolectomy in December 2016, and the 15 dissected lymph nodes did not reveal metastatic involvement. He did not require adjuvant chemotherapy or radiation and was deemed in remission. The resected bowel included a portion of small bowel that incidentally demonstrated pathological changes consistent with coeliac disease; however, he was negative for anti‐tissue transglutaminase antibodies with a normal immunoglobulin (Ig) A level. He underwent an oesophagogastroduodenoscopy that revealed duodenal inflammation and ulceration, villous atrophy, and intraepithelial lymphocytes, consistent with coeliac disease. He commenced a gluten‐free diet.

**Figure 1:**
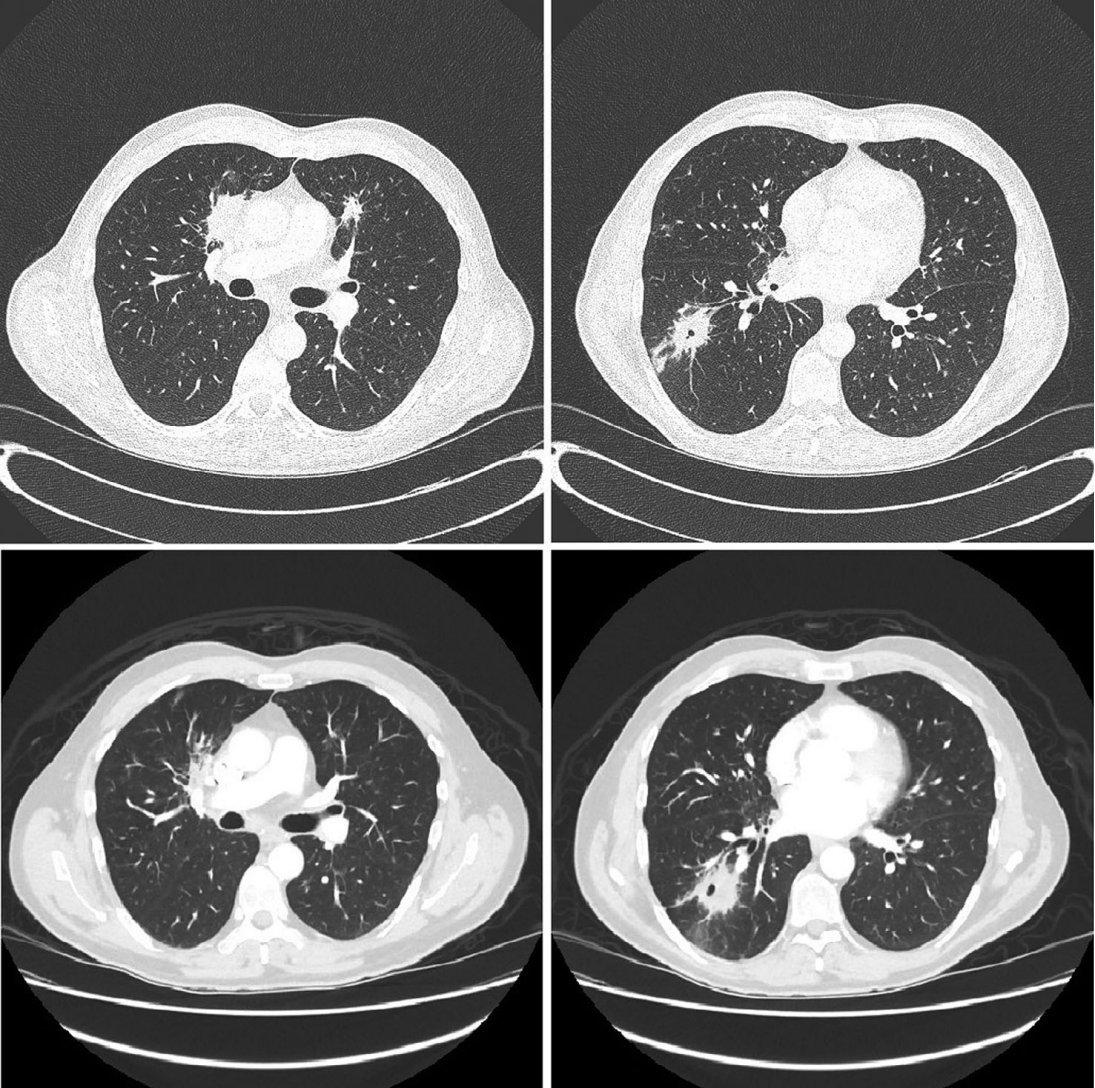
Lung high‐resolution computed tomography (HRCT). Multifocal consolidation and right lower lobe cavitary lung mass on presentation (top two panels) and after 10 weeks of prednisone (lower panels).

**Figure 2:**
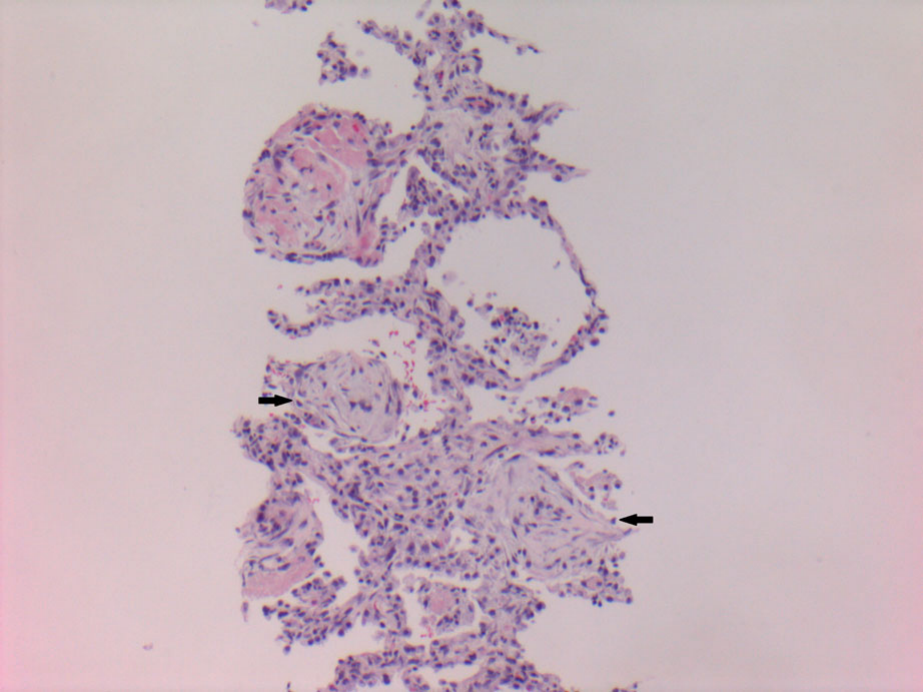
Transthoracic lung biopsy (haematoxylin and eosin (H&E) stain, 100× magnification) showing interstitial inflammation, pneumocyte hyperplasia, and alveolar fibrous plugs (arrows).

He was well until August 2017, when he was referred to our Respirology Department with a four‐month history of fever, productive cough, and intermittent haemoptysis despite four courses of antibiotics. HRCT revealed a 5‐cm dominant right lower lobe cavitary lesion, multifocal consolidation, and nodular infiltrates (Fig. 1). He underwent a bronchoscopy with radial endobronchial ultrasound‐guided transbronchial biopsies (TBBs) and one computed tomography (CT)‐guided transthoracic biopsy (TTB) of the mass. TBB revealed acute inflammation and TTB revealed organizing fibrous plugs with inflammatory cells, consistent with OP (Fig. 2). All biopsies, lymph node aspirates, bronchoalveolar lavages, and washes were negative for malignancy, granulomas, vasculitis, or infection, including Ziehl–Neelsen stains for acid‐fast bacilli and mycobacterial culture. Autoimmune serology was negative. He was started on prednisone 50 mg daily in October 2017, and due to symptomatic improvement and undesirable side effects such as insomnia, a taper was commenced after seven weeks on this dose. Unfortunately, in December 2017, he developed worsening cough, dyspnoea, and haemoptysis while on prednisone 20 mg daily. Repeat CT in December 2017 demonstrated improvement in multifocal consolidation and nodular infiltrates, and minimal change in the dominant right lower lobe lesion (Fig. 1). He underwent a bronchoscopy with biopsy of a mucosal lesion that demonstrated acute and chronic inflammation with small areas of necrotic debris. He was assessed for a surgical lung biopsy; however, the depth of the lesion would have required a large resection and this was thought not warranted given three prior biopsies with similar histological findings. Ultimately, he restarted prednisone 50 mg in January 2018. A post‐mortem review of the mucosal biopsy revealed atypical CD8+ lymphocytes with some phenotypic aberrations, suggestive of but insufficient for a diagnosis of malignancy.

In February 2018, despite being on a proton pump inhibitor, the patient was admitted with a massive upper gastrointestinal bleed. He underwent three more oesophagogastroduodenoscopies in one month with biopsies of duodenal ulcers, the pathological examination of which showed findings similar to previous, but now with a T‐cell population with aberrant phenotype. This was thought to be consistent with refractory coeliac disease type II (RCDII).

He subsequently developed fevers, splenomegaly, pancytopenia, hypertriglyceridaemia, and marked hyperferritinaemia that peaked at 14,957 μg/L (normal: 12–300 μg/L). Bone marrow biopsy showed evidence of haemophagocytosis and a monoclonal T‐cell population not conclusively diagnostic of T‐cell lymphoma. Positron emission tomography/CT did not demonstrate definitive evidence of malignancy. He was discharged after a one‐month hospitalization with haematology follow‐up, but was re‐admitted three months later with ongoing fevers, significant constitutional symptoms, and persistent features of haemophagocytic lymphohistiocytosis (HLH). He was started on etoposide for treatment of HLH, and repeat bone marrow biopsies were consistent with T‐cell lymphoma, likely EATL given his clinical history. Unfortunately, he continued to decline and became too unwell for chemotherapy, and passed away in the critical care unit.

## Discussion

Our current case is the first reported case of OP associated with EATL. Our review of the literature revealed only 10 English language reports of OP associated with lymphoma that specify the absence of prior anticancer treatment (Table 1) [4, 6, 7, 8, 9, 10, 11, 12, 13, 14]. There did not appear to be a clear predilection for aggressive or indolent lymphoma subtypes, although nine out of 10 prior reports described cases of B‐cell lymphoma. OP was diagnosed anywhere from 15 years prior to eight months after the diagnosis of the lymphoma; it is likely in some cases that the two may be unrelated. Three patients had multiple relapses or progression of OP despite ongoing corticosteroid therapy [6, 9, 14], which is unusual for OP, and perhaps suggests a concomitant or alternate diagnosis.

**Table 1:** Patients with OP associated with lymphoma.

Citation	Age/sex	Lymphoma subtype	Timing of OP relative to lymphoma diagnosis	HRCT findings	Mode of lung biopsy	Mode of lymphoma diagnosis	Lymphoma treatment	OP treatment and outcome
Romero, 1992 [6]	58/F	Diffuse mixed small and large cell lymphoma	Four years prior	Not available	TBB	Cervical node biopsy	CHOP; in remission	Ongoing relapses and steroid dependent; alive at two years
Safadi, 1997 [7]	44/M	T‐cell‐rich B‐cell lymphoma	Six months prior	Peripheral infiltrates	TBB, TTB; OLB six months later	OLB	CVP; partial response	Initial response to hydrocortisone; died at nine months from pneumonia/haemoptysis
Daniels, 2007 [4]	73/M	Cutaneous T‐cell lymphoma	Later	Diffuse infiltrates	VATS	Not available	Not available	No OP treatment, resolved independently, alive at 20 months
Paul, 2014 [8]	82/M	Lymphoplasmacytic lymphoma	Six weeks prior	Multiple nodular opacities	TTB; VATS six weeks later	VATS	Prednisone; good response	Improved on prednisone; unclear duration of follow‐up
Nedelcu, 2015 [9]	65/M	HL	Concomitant; HL diagnosed three months later	Mass‐like opacity	OLB	OLB	None	Progression on prednisone; died at three months from ACS
Polaczek, 2015 [10]	65/M	CLL progressing to diffuse mixed cell lymphoma	Eight months later	Localized consolidation and GGO	OLB	OLB	None	Complete resolution with prednisone; alive at nine months
Corina Sela, 2018 [11]	65/F	Marginal zone lymphoma	Concomitant	Pulmonary alveolar opacities	TBB, VATS	VATS	Rituximab; in remission	Complete resolution with corticosteroids; unclear duration of follow‐up
Lacerda, 2017 [12]	36/F	DLBCL	Concomitant	Multiple nodular foci of consolidation	TTB	Cervical node biopsy	R‐CHOP, RT; in remission	Complete resolution with R‐CHOP; unclear duration of follow‐up
Lal, 2018 [13]	20/F	HL	Concomitant; HL diagnosed four months later	Mass abutting left mediastinum	OLB ×2	OLB (second)	ABVD, RT; in remission	No recurrence after course of prednisone; alive at seven years
Bordas‐Martinez, 2020 [14]	56/M	HL and DLBCL	15 years prior; biopsy proven four years prior	Relapsing migratory lung infiltrates	VATS, cryobiopsy, autopsy	Psoas biopsy, autopsy*	Rituximab	Multiple relapses, steroid‐dependent; died from sepsis on rituximab
Current patient	61/M	Enteropathy‐associated T‐cell lymphoma	Nine months prior	Multifocal nodules and consolidation, cavitation	TBB, TTB	Duodenal biopsy	None	One relapse; died from HLH at nine months

* DLBCL diagnosed on psoas lesion biopsy; pulmonary involvement by HL and DLBCL seen only on autopsy.

ABVD, doxorubicin, bleomycin, vinblastine, and dacarbazine; ACS, acute coronary syndrome; CHOP, cyclophosphamide, doxorubicin, vincristine, and prednisone; CLL, chronic lymphocytic leukaemia; CVP, cyclophosphamide, doxorubicin, prednisone; DLBCL, diffuse large B‐cell lymphoma; GGO, ground‐glass opacification; HL, Hodgkin's lymphoma; HLH, haemophagocytic lymphohistiocytosis; HRCT, high‐resolution computed tomography; OLB, open lung biopsy; OP, organizing pneumonia; R‐CHOP, rituximab, cyclophosphamide, doxorubicin, vincristine, and prednisone; RT, radiation therapy; TBB, transbronchial biopsy; TTB, transthoracic biopsy; VATS, video‐assisted thoracoscopic surgery.

In seven patients, OP and lymphoma were diagnosed on the same biopsy specimen, with OP seemingly a direct reaction to lymphomatous infiltration of the lung [7, 8, 9, 10, 11, 13, 14]. In three of the four remaining cases (including our case), diagnosis of OP was made by TBB or TTB, making it difficult to exclude concurrent pulmonary lymphoma due to sampling error [6, 12]. Indeed, of the six patients who were initially diagnosed with OP via TBB or TTB, three also underwent a surgical lung biopsy. All three surgical lung biopsies revealed lymphomatous infiltration of lung tissue that was missed on initial biopsy [7, 8, 11], highlighting the shortcomings of minimally invasive biopsy techniques. In our case, the atypical features of OP (cavitation and haemoptysis) and the atypical T‐cell infiltrate on mucosal biopsy may suggest that OP may not have been the sole pulmonary pathology. Treatment of OP with prednisone may have partially treated an underlying lymphoma, lowering the diagnostic yield of duodenal, lung, and bone marrow biopsies. The diagnostic uncertainty in our case did prompt a multidisciplinary review to consider surgical lung biopsy; however, at that time, there was no suspicion of systemic, haematological, or malignant disease, and three non‐invasive biopsies with similar histological features provided us with enough confidence in the diagnosis of OP. Alternative diagnoses such as atypical infection or, more pertinently, a pulmonary lymphoma may have been missed. Alternatively, the OP may be completely unrelated to the lymphoma, that is, a cryptogenic OP.

The mechanism linking OP and lymphoma is unclear; however, several mechanisms have been suggested in prior literature [7, 10, 13]. We hypothesize that OP and lymphoma could be two results of a common immune phenomenon that has not yet been elucidated. OP is classically thought to be fibroblast‐mediated; however, some studies suggest a role of T‐cells in its pathogenesis [15, 16, 17]. Certainly, RCDII, EATL, and HLH represent syndromes of major T‐cell dysfunction, perhaps precipitated initially by coeliac disease and formation of a dysfunctional T‐cell clone. The presence of atypical lymphocytes in our patient's duodenal and bone marrow biopsies months before the diagnosis of lymphoma is supportive of an underlying T‐cell‐mediated phenomenon. In addition, given RCDII is considered a low‐grade intraepithelial lymphoma and frequently transforms into EATL [18], it is possible that OP developed around the time of progression. Polaczek et al. have previously described a similar case of a 65‐year‐old man with a history of chronic lymphocytic leukaemia (CLL) who was diagnosed with OP on an open lung biopsy that also revealed malignant progression to diffuse mixed cell lymphoma [10].

Prior studies have additionally proposed that OP could represent an inflammatory response to a pulmonary lymphoma that was missed due to sampling error, particularly in cases where minimally invasive techniques were utilized. Malignant infiltration of the lungs could cause obstruction of airways, giving rise to a reactive process that could include features of OP. Another proposed mechanism is that OP could represent a paraneoplastic syndrome preceding the clinical expression of a lymphoma that manifests months to years later. Lastly, in cases where OP preceded the diagnosis of a malignant pulmonary lesion, authors have postulated that OP (specifically, the involved lymphocytes) could undergo malignant transformation, although the variable time course of disease presentation in the literature makes this mechanism seem less likely [7, 10, 13].

In summary, OP may be seen in association with lymphoma, and should not immediately be attributed to anticancer treatment. The mechanism linking OP and lymphoma is unclear but OP could be the result of underlying T‐cell dysfunction or represent a direct reaction to malignant infiltration of the lung. In patients with atypical presentations, exclusion of an alternate diagnosis, including pulmonary lymphoma, must be pursued. The mode of lung biopsy should be made on a case‐by‐case basis, taking into account the sampling error demonstrated with TBB and TTB, but also considering the morbidity of surgical lung biopsy in patients with significant underlying comorbidities.

### Disclosure Statement

Appropriate written informed consent was obtained for publication of this case report and accompanying images.
